# Estimating epidemic dynamics with genomic and time series data

**DOI:** 10.1098/rsif.2024.0632

**Published:** 2025-06-04

**Authors:** Alexander E. Zarebski, Antoine Zwaans, Bernardo Gutierrez, Louis du Plessis, Oliver G. Pybus

**Affiliations:** ^1^ Department of Biology, University of Oxford, Oxford, Oxfordshire, UK; ^2^ School of Mathematics & Statistics, University of Melbourne, Melbourne, Victoria, Australia; ^3^ Department of Biosystems Science and Engineering, ETH Zurich, Basel, Switzerland; ^4^ Swiss Institute of Bioinformatics, Lausanne, Vaud, Switzerland; ^5^ Department of Pathobiology and Population Sciences, Royal Veterinary College, London, UK

**Keywords:** phylodynamics, genetic epidemiology, computational statistics, birth-death processes

## Abstract

Accurately estimating the prevalence and transmissibility of an infectious disease is an important task in genetic infectious disease epidemiology. However, generating accurate estimates of these quantities, that make use of both epidemic time series and pathogen genome sequence data, is a challenging problem. Phylogenetic birth–death processes are a popular choice for modelling the transmission of infectious diseases, but it is difficult to estimate the prevalence of infection with them. Here, we extended our approximate likelihood approach, which combines phylogenetic information from sampled pathogen genomes and epidemiological information from a time series of case counts, to estimate historical prevalence in addition to the effective reproduction number. We implement this new method in a BEAST2 package called Timtam. In a simulation study our approximation is seen to be well‐calibrated and recovers the parameters of simulated data. To demonstrate how Timtam can be applied to real datasets, we carried out empirical analyses of data from two infectious disease outbreaks: the outbreak of SARS-CoV-2 onboard the *Diamond Princess* cruise ship in early 2020 and poliomyelitis in Tajikistan in 2010. In both cases we recover estimates consistent with previous analyses.

## 1. Introduction

In the field of genetic infectious disease epidemiology, two key questions are commonly asked: ‘how many people are infected?’ (i.e. what is the prevalence?) and ‘how transmissible is this pathogen?’ (i.e. what is its effective reproduction number?). Prevalence of infection is the number of individuals infected at a given time and the effective reproduction number is defined as the average number of secondary infections per infectious individual at a given time.

Birth–death processes are a popular model for describing the transmission of infectious diseases; they capture the mechanism at an individual level and are amenable to analysis [1]. In the birth–death process, births represent new infections, and deaths, the end of an infectious period. Nee *et al*. [2] demonstrated how the branch lengths of a phylogeny can be used to estimate birth and death rates (of species), and Stadler *et al*. [3,4] demonstrated how this idea can be applied to the analysis of phylogenies of pathogen genomes. In Bayesian phylogenetics, the birth–death process enters the analysis as a prior distribution for the reconstructed phylogeny, the so-called *tree prior*.

The joint analysis of multiple data sources (e.g. sequenced and unsequenced data) is a long-standing challenge for infectious disease modelling [5], partly due to the difficulty of accounting for dependencies between datasets and weighing the information they provide. In previous work, we approximated a joint distribution that accounts for this dependency and relies on the distribution to weight the contributions of each dataset properly [6]. Assuming conditional independence of datasets is another approach that simplifies matters [7] but can present its own challenges [8].

Several methods can estimate the population size (i.e. prevalence of infection) using birth–death models. Andréoletti *et al*. [9], building on the work of Manceau *et al*. [10], numerically solved large systems of differential equations to evaluate the likelihood function needed to estimate prevalence. However, this becomes computationally intractable for even moderately sized datasets. In previous work, we described an efficient and accurate approximation of that likelihood function [6]. The approximation makes it possible to estimate both the basic reproduction number and the prevalence of infection at the present (but not the historical prevalence). In the current work, we describe a novel extension of our previous algorithm, which enables estimates of the prevalence throughout the epidemic; a software package implementing this for BEAST2; and preliminary steps towards extending the algorithm to handle sampled ancestors [11].

An alternative to handling the likelihood analytically, which has received substantial attention, is the use of particle filters. Particle filters simplify the likelihood in exchange for additional simulation and are used in particle MCMC methods [12]. For example, Rasmussen *et al*. [13] combined coalescent models, with a particle filter and a simplifying assumption of conditional independence between data sources; this work was extended by Rasmussen *et al*. [14] and Li *et al*. [15] to include the structured populations of epidemiological models. There is work underway to overcome this independence assumption in the context of coalescent models [16].

Particle filters have also been used to carry out inference with birth–death models. Vaughan *et al*. [17] presented the EpiInf BEAST2 package, however this exact method is intractable for larger datasets. More recently, Judge *et al*. [7] presented the EpiFusion software package, which uses a conditional independence assumption (like Rasmussen *et al*. [14]) to allow it to scale to larger datasets. In benchmarks, EpiFusion was found to outperform both EpiInf and Timtam in terms of estimate accuracy but cannot estimate the phylogenetic tree and instead requires it to be provided as a model input [7]. While particle filters offer a great deal of flexibility, they can be computationally expensive and, while we have a range of diagnostic methods to assess the quality of MCMC samples, the diagnostics for particle filters are less well developed.

We have implemented the extended approximation in a BEAST2 package called Timtam. This package makes it possible for phylogenetic analyses to make use of time series data and estimate the prevalence of infection, while leveraging the functionality provided by the rest of the BEAST2 ecosystem, e.g. it supports time-varying parameters and a suite of substitution and molecular clock models. Timtam can be downloaded from CBAN, the BEAST2 package repository, and there are several tutorials included with the software https://github.com/aezarebski/timtam2.

In an outbreak of infectious disease, typically, only a small number of cases have a genome of the pathogen sequenced. For example, no country with a sizeable COVID−19 outbreak sequenced >20% of reported cases and most sequenced <5%. Among low and middle-income countries this number is often <1%. However, unsequenced case data is also informative and can help to refine estimates of epidemic parameters [7,13]. Timtam makes it computationally feasible to analyse both types of data simultaneously for large outbreaks [6].

We carried out a simulation study to demonstrate that our methodology leads to well-calibrated estimates, i.e. that approximately 95% of the 95% highest posterior density intervals (HPD intervals, i.e. the credible intervals) contain the true parameter value from the simulation. We further demonstrate the ‘real-world’ use of this package with two empirical case studies. In the first, we repeat an analysis by Andréoletti *et al*. [9] of SARS-CoV-2 data from an outbreak onboard the *Diamond Princess* cruise ship. We compare our estimates of the reproduction number and the prevalence to similar analyses of this outbreak [9,18]. The prevalence estimates from Andréoletti *et al*. [9] can only be interpreted as lower bounds due to the limitations of their algorithm, and the results from Vaughan *et al*. [18] were obtained using a computationally expensive simulation-based approach. In the second, we reanalyse data from the 2010 outbreak of poliomyelitis in Tajikistan [19,20] and compare the results to a similar analysis [15]. Our analysis includes novel estimates of the prevalence of infection.

## 2. Methods

**Figure 1.**
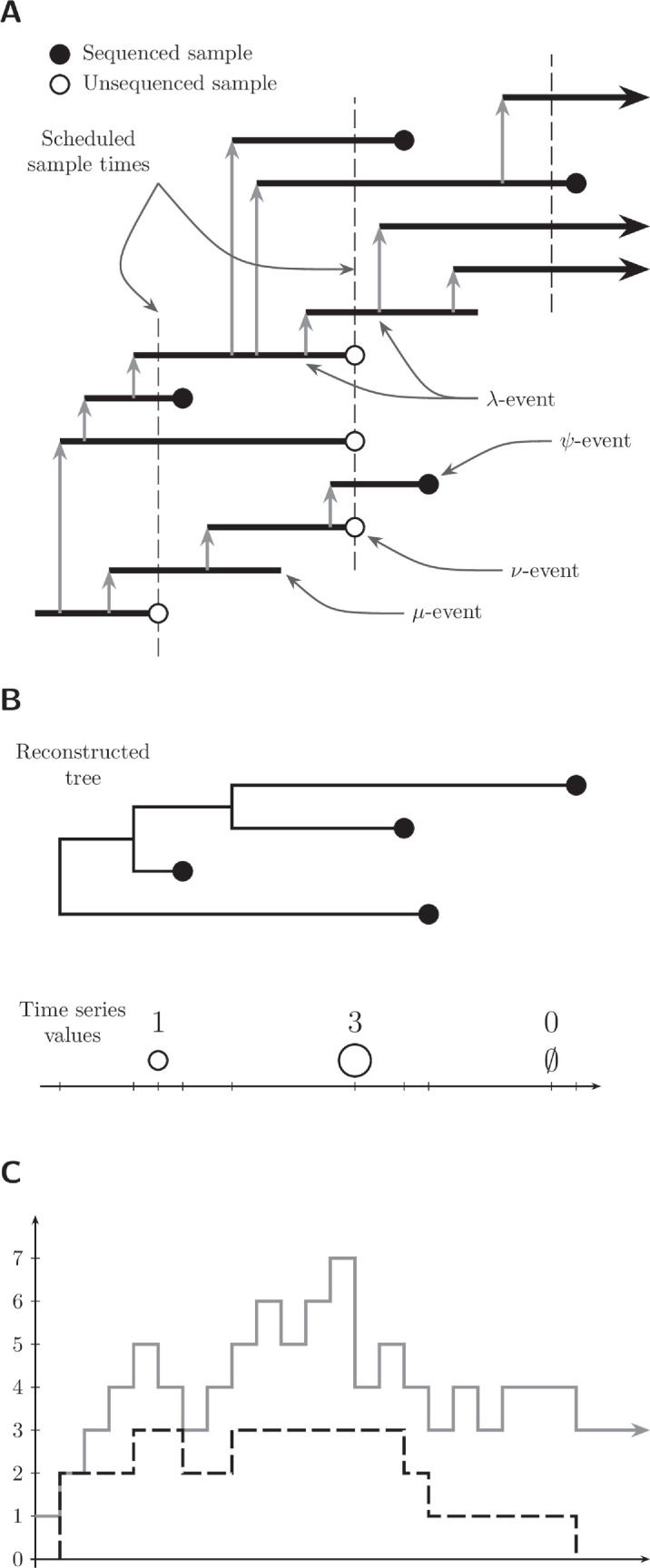
The transmission process is viewed as a sequence of events with the observations processed sequentially to approximate their joint likelihood. (A) Transmission tree with intervals of time an individual was infected indicated by horizontal lines and the vertical grey arrows indicating transmission. Three scheduled unsequenced samples are taken at the times indicated by the vertical dashed lines. (B) Corresponding reconstructed tree and time series of confirmed cases in each of the scheduled unsequenced samples. In the third sample no cases were observed. (C) Prevalence of infection (grey line) and the lineages through time (LTT) plot (black dashed line).

Figure 1 demonstrates the different views of an epidemic that are used in phylodynamics and will help us to establish some terminology for discussing our model. Figure 1A shows a transmission tree, a complete description of who-infected-whom during an epidemic, the timing of these events and the observations of this process. In the transmission tree each infection is represented by a horizontal line indicating the infectious period with grey arrows indicating when someone has transmitted the infection. An infectious period can end in one of three ways in this example: sequenced infections end in filled circles, observed (but unsequenced) infections end in unfilled circles and unobserved infections end without a circle. Ongoing infections are indicated with arrows. In this example, the unobserved end of infection occurs at rate μ and the sampling of sequenced infections occurs at rate ψ. Since the sequenced samples follow a point process, we refer to them as *unscheduled* data. The vertical dashed lines indicate times at which a subset of infected individuals are observed as cases, we refer to this as *scheduled* data. Since time series of cases are a ubiquitous representation, it is useful for the process to be able to model this data.

Figure 1B shows the reconstructed tree and time series of case counts from the transmission tree in figure 1A. The reconstructed tree is the subtree of the transmission tree which has only the sequenced samples. The leaves of the transmission tree corresponding to unsequenced samples form a separate, but not independent, time series of confirmed cases.

The information in the reconstructed tree can be summarized by a lineages through time (LTT) plot. The *prevalence* of infection at time t is the number of infected individuals in the whole transmission tree at time t. Figure 1C shows the LTT for the reconstructed tree as a dashed line and the prevalence as a solid line. The prevalence of infection may count lineages not descended from the MCRA of the sequenced samples. We refer to the lineages in the transmission tree that are not in the reconstructed tree as the *hidden lineages* because they are not visible in the data. We denote by $k_{t}$ the value of the LTT at time t and by $H_{t}$ the number of hidden lineages at time t. The prevalence at time t is $k_{t}+H_{t}$.

Figure 1 shows unscheduled sequenced data and scheduled unsequenced data; however, we may also consider arbitrary combinations of (un)sequenced and (un)scheduled observations. In this manuscript we focus on datasets with unscheduled sequenced data and scheduled unsequenced data, i.e. time-stamped sequences and a time series of confirmed but not sequenced cases, since this aligns closest to typical epidemiological datasets.

### 2.1. Likelihood function

In an epidemiological setting, we are often interested in the prevalence of infection and the effective reproduction number, $R_{\text{e}}(t)$, because these quantities are of critical importance when assessing the threat posed by an outbreak of infectious disease. Bayesian phylodynamic methods provide a coherent solution with a clear quantification of uncertainty. This usually requires us to evaluate the joint posterior distribution of the model parameters and the reconstructed tree (up to an unknown normalization constant if we are using MCMC to generate posterior samples), conditioning on time-stamped viral genomes and a time series of confirmed cases.

The data we condition upon consists of $D_{\text{MSA}}$ and $D_{\text{cases}}$, where $D_{\text{MSA}}$ is the multiple sequence alignment (MSA) containing the time-stamped pathogen genomic data, and $D_{\text{cases}}$ is the observation of confirmed cases without associated pathogen genomes.

The parameters of this process partition into four groups:

— H, the number of hidden lineages at specified points in time (which we use to estimate the prevalence of infection);
— T, the time-calibrated reconstructed tree describing the ancestral relationships between the sequences in $D_{\text{MSA}}$;
— $\theta_{\text{evo}}$ the parameters of the evolutionary model, describing how genome sequences change over time (e.g. the molecular clock rate and nucleotide substitution model relative rate parameters);
— and $\theta_{\text{epi}}$, the parameters of the epidemiological model, describing how the outbreak/epidemic grows or declines over time and how we observe it.

Using the terminology of birth–death processes, $\theta_{\text{epi}}$ contains the birth rate λ and the death rate μ along with the sequenced sampling rate ψ, the unsequenced sampling rate ω (a.k.a. the occurrence rate), the probability of observation in a scheduled sequenced sample ρ and the probability of observation in a scheduled unsequenced sample ν. Examples of these events are shown in figure 1A. Throughout this manuscript, we treat these parameters as piecewise constant functions with known change times.

We can express the posterior distribution, $f\;(H,T,\theta_{\text{epi}},\theta_{\text{evo}}|D_{\text{MSA}},D_{\text{cases}})$, in terms of simpler components as in equation (2.1). The likelihood of the sequence data given the reconstructed tree and genomic parameters $f\;(D_{\text{MSA}}|T,\theta_{\text{evo}})$, which appears in equation (2.1), is the *phylogenetic likelihood*. This can be efficiently calculated with Felsenstein’s pruning algorithm [21]. The likelihood of the time series of cases, reconstructed tree and prevalence, given the epidemiological parameters $f\;(D_{\text{cases}},T,H|\theta_{\text{epi}})$, often called the *tree prior*, is more accurately called the *phylodynamic likelihood*. Here, we make the standard simplifying assumption that there is no dependence between the tree structure and the sequence evolutionary process. Consequently the phylogenetic likelihood is independent of $D_{\text{cases}},H$ and $\theta_{\text{epi}}$. We also assume $\theta_{\text{epi}}$ and $\theta_{\text{evo}}$ have independent priors.


(2.1) $$f\;(H,T,\theta_{\text{epi}},\theta_{\text{evo}}|D_{\text{MSA}},D_{\text{cases}})=\frac{\underset{\text{phylogenetic likelihood}}{\underbrace{f\;(D_{\text{MSA}}|T,\theta_{\text{evo}})}}\underset{\text{phylodynamic likelihood}}{\underbrace{f\;(D_{\text{cases}},T,H|\theta_{\text{epi}})}}f\;(\theta_{\text{epi}})f\;(\theta_{\text{evo}})}{f\;(D_{\text{MSA}},D_{\text{cases}})}.$$

The technical details of the method used to approximate the phylodynamic likelihood can be found in our previous work [6]. These details are also given in §2 of the electronic supplementary information. In this paper, we present two novel extensions to that existing methodology. The first is the inclusion of the prevalence as a random variable under the posterior distribution, which enables us to estimate the prevalence of infection at specific times (as described in §2.1 of the electronic supplementary information). The second is derivations of the expressions needed to account for both sampled ancestors for unscheduled observations and scheduled sequenced observations. The details of these extensions can be found in §4 of the electronic supplementary information.

#### 2.1.1. The effective reproduction number

We define the effective reproduction number $R_{\text{e}}(t)$ as the total expected number of secondary infections generated by an individual newly infected at time t. When there is no scheduled sampling, $R_{\text{e}}=\lambda /(\mu +\psi +\omega )$. Calculating $R_{\text{e}}$ becomes complicated when there is scheduled sampling because there is a combination of continuous resolution of infection and instantaneous removal due to sampling. By separating the infections caused before and after the next scheduled sample, we obtain recursive expressions for $R_{\text{e}}$. These expressions are derived in §3 of the electronic supplementary information. Since all individuals observed in scheduled samples simultaneously cease to be infectious this leads to a jump in $R_{\text{e}}$ at each scheduled sample event as some infections are cut short. Calculating $R_{\text{e}}$ is non-trivial (from a computational perspective), so we use the following approximation of it instead.

Consider data consisting of unscheduled sequences and scheduled unsequenced samples at regular intervals of duration $\Delta_{t}$, i.e. a point process of sequenced samples and a time series of confirmed cases. From the perspective of an infectious individual, if we condition upon their being removed in a scheduled sample, the number of samples until this occurs, W, has a geometric distribution with probability ν. Given the scheduled samples occur at regular intervals of duration $\Delta_{t}$, the waiting time is approximately $\Delta_{t}(W+1/2)$ (where the half follows from a continuity correction). The rate of an exponential distribution with the same mean is $2\nu /(2\Delta_{t}-\nu \Delta_{t})$.

This suggests the following approximation: we replace the scheduled unsequenced sampling with unscheduled unsequenced sampling at a rate $\tilde{\omega }=2\nu /(2\Delta_{t}-\nu \Delta_{t})$, i.e. we approximate the scheduled sampling with unscheduled sampling at a comparable rate, $\tilde{\omega }$. The rate is obtained by matching the geometric and exponential distributions as described above. With unscheduled unsequenced sampling at rate $\tilde{\omega }$ the expression for $R_{\text{e}}$ is $\lambda /(\mu +\psi +\tilde{\omega })$.

**Figure 2.**
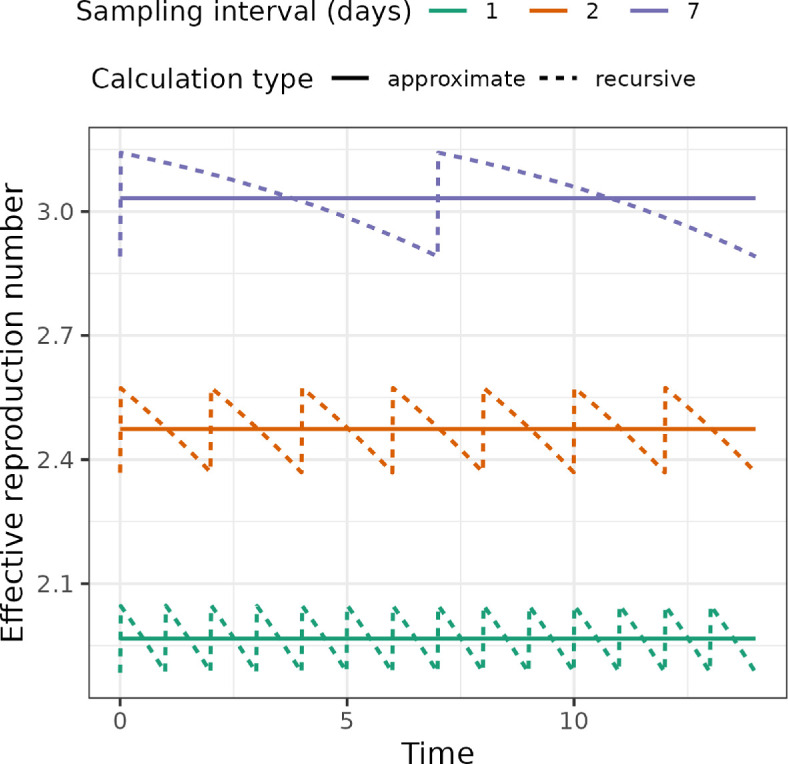
The effective reproduction number through time and its approximation. The approximation smooths out the saw-tooth value of the (recursively computed) effective reproduction number, which occurs when there are scheduled samples. The parameters used for this figure are birth rate of 0.4, death rate of 0.1, sampling rate of 0.02 and a scheduled unsequenced sampling probability of 0.08 (at varying intervals). The solid lines indicate the values obtained with our approximation and the dashed lines indicate the true values accounting for scheduled sampling.

Figure 2 shows the effective reproduction number calculated using both the recursive method described in §3 of the electronic supplementary information and the approximation in terms of $\tilde{\omega }$. The values of $R_{\text{e}}$ are greater for longer intervals between scheduled samples, $\Delta_{t}$, because there is (on average) a longer duration during which the individual can infect others before being removed in a scheduled sample.

The distribution of $\Delta_{t}(W+1/2)$ limits to an exponential distribution as $\Delta_{t}$ and ν go to zero (with a fixed ratio). Hence, we expect this approximation to work well when these values are small, i.e. when there is frequent sampling with low ascertainment. In the simulation study described below, we found the estimates obtained with and without the approximation are similar, suggesting it will work in practice.

#### 2.1.2. Model parameterizations

There are multiple ways to parameterize the birth–death–sampling process. We refer to the parameterization in terms of the rates λ, ψ, ω and probabilities ρ, and ν as the *canonical parameterization*. This parameterization is convenient mathematically, and we derive the approximate likelihood in terms of these parameters. In an epidemiological context, where we are typically faced with a time series of confirmed cases and point process sequence data, we prefer an alternative parameterization, the *time series parameterization*. This parameterization is in terms of the effective reproduction number, $R_{\text{e}}$, the net removal rate of infectious individuals, $\sigma =\mu +\psi +\tilde{\omega }$ (where $\tilde{\omega }$ is as described above), the probability that an infection appears in the time series, $\tilde{\omega }/\sigma$, and the probability that an infection is sequenced, ψ/σ. Note that when we use the time series parameterization in the SARS-CoV-2 and poliomyelitis analyses, we use the approximation of $\tilde{\omega }$ to simplify the model specification.

## 3. Results

### 3.1. Simulation study

To assess whether Timtam is a well-calibrated model and to evaluate the validity of the $R_{\text{e}}$ approximation, we carried out a simulation study. We simulated 100 epidemics from a birth–death process using remaster [22]. Each epidemic ran for 56 (simulated) days with the birth rate decreasing on day 42, (i.e. boom-bust dynamics), and two types of surveillance: sequenced and unsequenced with fixed rates, (see table 1.) We assume a known removal (death) rate. The prevalence of infection in each of the simulated epidemics is shown in electronic supplementary material, figure S1. There is a substantial amount of variability in the prevalence across the simulations, but the boom-bust dynamics can be seen in the average of the simulation trajectories.

**Table 1.** The simulated data for the calibration study was sampled from a birth-death process with two types of sampling and a change in birth rate leading to `boom-bust' dynamics. A final sequence sample is collected at the end of the simulation to ensure a consistent duration across the 100 replicates. Each simulation was conditioned to have at least two sequenced samples and a positive final prevalence of infection.

event	rate	transition
infection	λ(t)	$X\overset{\lambda }{⟶}2X$
removal	μ=0.046	$X\overset{\mu }{⟶}\emptyset$
sequence	ψ=0.008	$X\overset{\psi }{⟶}\text{Sequence}$
occurrence	ω=0.046	$X\overset{\omega }{⟶}\text{Case}$

We assume a known death rate, μ=0.046. The number of infectious individuals, X , was simulated for 56 days (after starting with a single infection x(0)=1). The birth rate is λ(t)=0.185 for *t* < 42 (‘boom’: Re=1.85) and λ(t)=0.0925 for t≥42 (’bust’: Re=0.925).

From each simulation we constructed two datasets: one with unsequenced samples treated as a point process, and a second with these samples aggregated into a time series of daily case counts. The parameters used are similar to those used in a previous simulation study [6] with an extension for the change in birth rate; we based the parameter values on the early dynamics of the SARS-CoV-2 epidemic in Australia. The code implementing this simulation and the subsequent inference is available at https://github.com/aezarebski/timtam-calibration-study.

**Figure 3.**
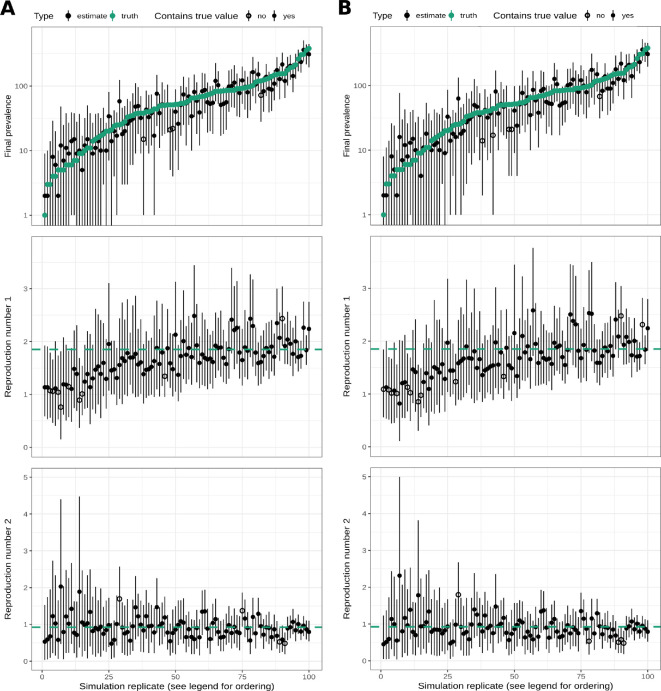
Parameter estimates converge to true values as the dataset gets larger. The solid black lines display the HPD intervals, and points indicate the point estimates; the point is filled if the HPD interval contains the true value and empty if it does not. The green points and the green dashed lines indicate the true values of the final prevalence and the reproduction number in the boom and bust portions of the simulation. We ordered the replicates by the final prevalence in each simulation. (A)The estimates when both sequenced and unsequenced data are treated as a point process. (B) The estimates when the unsequenced observations were aggregated into a time series of daily case counts.

We sampled the posterior distribution of the model parameters for each simulated dataset using the Timtam package and compared the resulting estimates to the true values from the simulations. Figure 3A shows the point estimates and 95% highest posterior density (HPD) intervals for the final prevalence and reproduction numbers across the simulations, ordered by the final prevalence in the simulation, in the case where the unsequenced data is modelled as a point process. Figure 3B shows the corresponding results when the unsequenced data are aggregated into a daily time series of counts.

As shown in electronic supplementary material, figure S2, there is a strong correlation between the final prevalence and the total number of observed cases (i.e. the size of the dataset.) The estimates shown in figure 3 suggest that in the simulations with a greater final prevalence, the resulting estimates have a smaller error and less uncertainty. Investigating further, electronic supplementary material, figure S3 shows a scatter plot of the error in the point estimates and the width of the 95% HPD intervals for the reproduction number, plotted against the size of the corresponding dataset. There is a trend of decreasing error and uncertainty as the dataset grows.

We tested the null hypothesis that 95% of the HPD intervals contain the true parameter, i.e. the model is well‐calibrated. The truth of this null depends upon the choice of prior distribution, nonetheless, we would like it to be difficult to falsify such a null hypothesis for plausible prior distributions. In our hypothesis test, we expect 91−99 of the HPD intervals to contain the true parameter value (out of the total 100 replicates).

Table 2 contains a summary of the rate parameter prior distributions and estimates from the first set of simulations (i.e. the ones with point process data) and table 3 contains the corresponding summary for the second set of simulations (i.e. with unsequenced samples aggregated into a time series). For the estimates based on the point process data, the HPD intervals of both the reproduction number and the prevalence at the time of the last sequenced sample have a coverage that is consistent with the desired level (95%). This suggests the estimation method is well‐calibrated. For the estimates based on the aggregated data, the coverage for $R_{\text{e}}^{1}$ is lower than desired, however for both $R_{\text{e}}^{2}$ and H the coverage is suitable. Similarly, the coverage is suitable for the process parameter estimates: birth, death and sampling rates. This suggests that, despite the model misspecification due to aggregating the point process data, we are still able to generate good HPD intervals for the reproduction number and prevalence.

**Table 2.** Posterior parameter estimates and accuracy in the 100 simulations. There are boom-bust dynamics, for the first 42 days of the simulation the birth rate is $\lambda_{1}$ after which it changes to $\lambda_{2}$ for the subsequent 14 days. The death rate is assumed known. When a prior distribution depends upon the birth-death process and the other parameters, we use `by process' to describe its prior distribution.

par	truth	prior	median	error	bias	width	coverage
$\lambda_{1}$	0.185	lognormal(−2.0, 1.0)	0.186	0.116	0.004	0.523	94
$\lambda_{2}$	0.092	lognormal(−2.0, 1.0)	0.095	0.337	0.032	1.375	94
μ	0.0460	—	—	—	—	—	—
ψ	0.008	lognormal(−3.0, 1.0)	0.010	0.351	0.275	1.754	96
ω	0.046	lognormal(−2.0, 1.0)	0.052	0.248	0.140	1.163	98
$R_{\text{e}}^{1}$	1.850	by process	1.689	0.180	–0.087	0.664	91
$R_{\text{e}}^{2}$	0.925	by process	0.897	0.291	–0.030	1.132	96
H	—	by process	—	0.360	–0.046	—	97

For each parameter (Par), the true value, the prior used, the median over the 100 medians of the estimate, relative error, relative bias, relative width and the percentage of HPD intervals containing the true value is provided.

**Table 3.** Posterior parameter estimates and accuracy in the 100 simulations after we aggregated the unsequenced observations into daily counts and used the resulting time series as data. See table 2.

par	truth	prior	median	error	bias	width	coverage
$\lambda_{1}$	0.185	lognormal(−2.0, 1.0)	0.186	0.121	0.003	0.535	91
$\lambda_{2}$	0.092	lognormal(−2.0, 1.0)	0.094	0.337	0.018	1.377	95
μ	0.0460	—	—	—	—	—	—
ψ	0.008	lognormal(−3.0, 1.0)	0.010	0.344	0.267	1.757	96
$\tilde{\omega }$	0.046	ν∼Uniform(0.0,1.0)	0.053	0.265	0.143	1.185	98
$R_{\text{e}}^{1}$	1.850	by process	1.670	0.191	–0.097	0.655	88
$R_{\text{e}}^{2}$	0.925	by process	0.873	0.287	–0.057	1.141	95
H	—	by process	—	0.367	–0.055	—	96

### 3.2. SARS-CoV-2 on the Diamond Princess cruise ship

**Figure 4.**
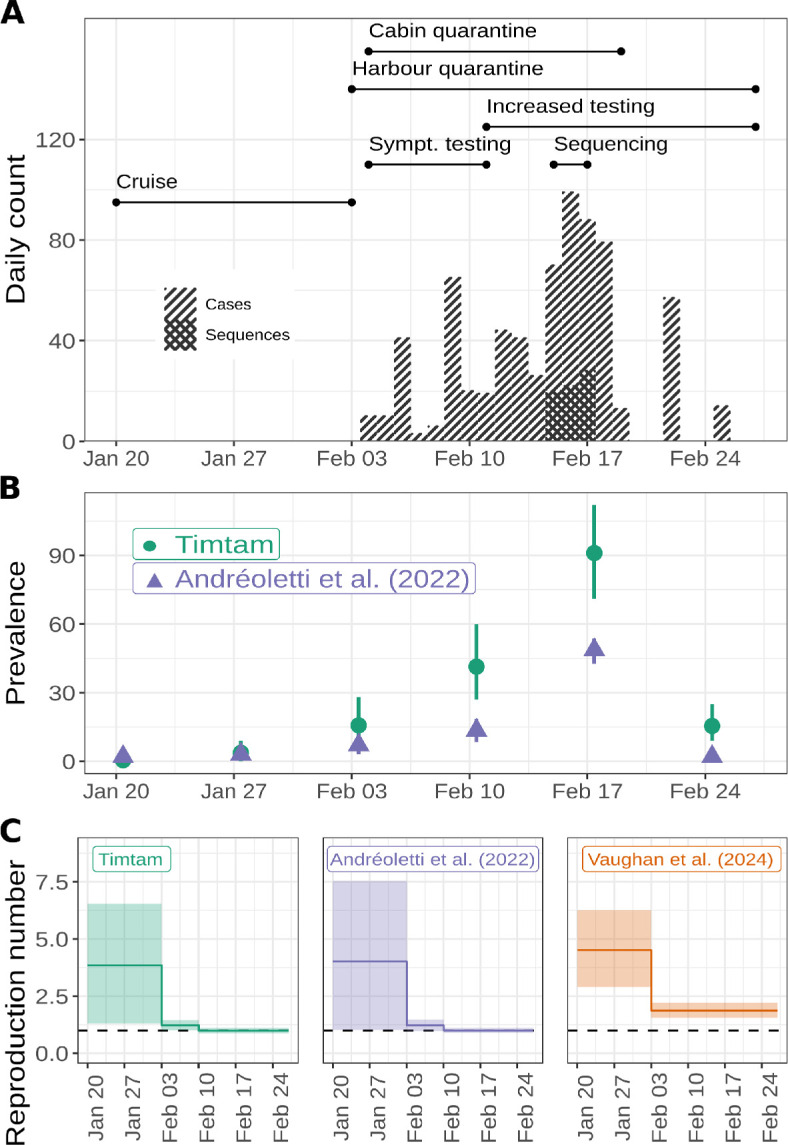
SARS-CoV-2 aboard the *Diamond Princess* cruise ship. (A) Sequences were collected across three days and testing varied throughout the quarantine period. The stacked bar chart shows the daily number of confirmed cases and sequenced samples. We indicate the timing of changes to surveillance and quarantine with lines at the top of the figure. (B) Estimates of the prevalence of infection and the 95% HPD intervals onboard the *Diamond Princess*. In addition to our estimates (green) estimates from Andréoletti *et al*. [9] are shown (purple). (C) Estimates of the reproduction number and the 95% HPD intervals. In addition to our estimates (shown in green) estimates from Andréoletti *et al*. [9] (purple) and Vaughan *et al*. [18] (orange) are shown.

To demonstrate the utility of our new approach we replicated an analysis of a SARS-CoV−2 outbreak onboard the *Diamond Princess* cruise ship [9]. This outbreak is particularly well suited to analysis because it occurred on an isolated cruise ship (with 3711 people onboard) in a carefully monitored population with detailed accounts of isolation and testing measures. The outbreak appears to have originated from a single introduction of the virus [23]. Figure 4A displays the cases and sequencing effort across the duration of the quarantine. We obtained a time series of daily confirmed cases [24] to use as $D_{\text{cases}}$ and an alignment of 70 pathogen genomes [23] was used as $D_{\text{MSA}}$. The accession numbers for the sequences are available in §6 of the electronic supplementary information.

#### 3.2.1. Model

We modelled the sequenced SARS-CoV-2 infections as a point process, consistent with previous analyses of the data. Where multiple samples were available for a particular day, we uniformly spaced the sequenced samples across the day the samples were collected. A more nuanced analysis would have modelled these samples as scheduled sequenced samples; however, this would make the resulting estimates harder to compare to previous results and complicate the interpretation.

We made minor adjustments to the model to better match standard epidemiological workflows for $R_{\text{e}}$ estimation, as described in §6 of the electronic supplementary information. Importantly, we modelled daily case counts of confirmed cases as scheduled samples (i.e. a time series) instead of unscheduled samples (i.e. a point process of occurrences) electronic supplementary material, table S1 lists the prior distributions used in the model. The XML file specifying the analysis and post-processing scripts is available from https://github.com/azwaans/timtam-diamond-princess.

#### 3.2.2. Results

Figure 4B shows the estimates of the prevalence of infection and the 95% HPD intervals along with the corresponding values from Andréoletti *et al*. [9]. Our estimates suggest a larger prevalence of infection than the estimates from Andréoletti *et al*. [9]. As discussed below, we attribute this difference in the prevalence estimates to their implementation having an upper limit of 40 hidden lineages. We do not have estimates of prevalence from the analysis by Vaughan *et al*. [18] as they estimated the cumulative number of infections, instead of the prevalence.

Figure 4C shows the estimates of the reproduction number through time along with the 95% HPD intervals. The estimates of the effective reproduction number are consistent with those from previous analyses of these data by Andréoletti *et al*. [9]. Our estimates differ from those of electronic supplementary material, figure S4 Vaughan *et al*. [18]. This discrepancy may be due to the different datasets used: our analysis used both the time series of confirmed cases and pathogen genomes, while Vaughan *et al*. [18]’s estimates are based on genomic data alone.

**Figure 5.**
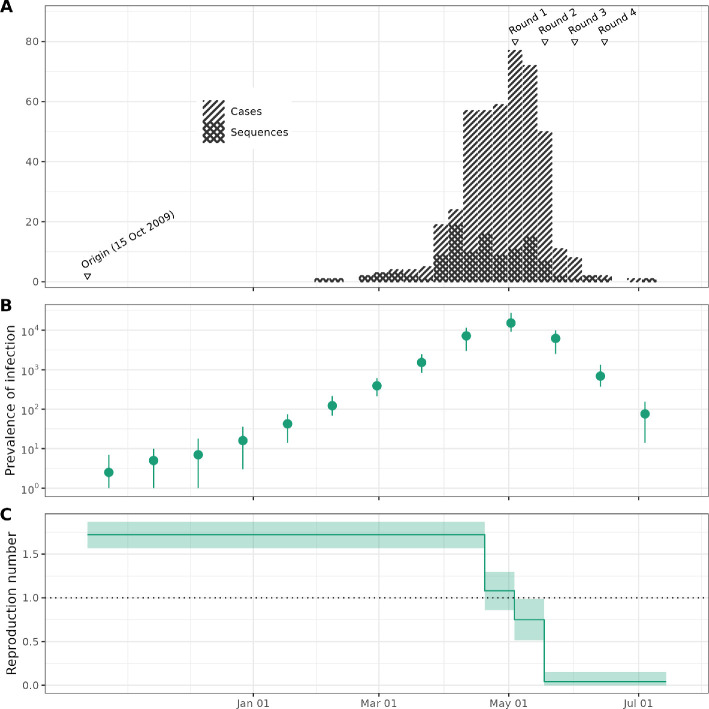
Poliomyelitis in Tajikistan in 2010. (A) Sequences were collected throughout the outbreak. The stacked bar chart shows the weekly number of confirmed cases and sequenced samples. We indicate the hypothesized origin time and the timing of vaccination rounds at the top of the figure. (B) Estimates of the prevalence of infection (on a logarithmic scale) and the 95% HPD intervals at 21 day intervals across the outbreak. (C) Estimates of the reproduction number and the 95% HPD intervals as constants before and after the start of vaccination.

### 3.3. Poliomyelitis in Tajikistan

Poliomyelitis (polio) is caused by infection with the poliovirus, an RNA virus spread through the faecal-oral route. While most poliovirus infections are asymptomatic, it has the potential to cause permanent paralysis. Polio has a long history but since the introduction of vaccines in the 1950s incidence has declined and there are sustained efforts towards eradication.

In 2010 there was an outbreak of wild poliovirus type 1 (WPV1) in Tajikistan. We reanalysed the genomic and time series data collected during the outbreak. These data had previously been jointly analysed by Li *et al*. [15] using an age-structured model [25]. A previous genomic analysis by Yakovenko *et al*. [19] suggests the outbreak of WPV1 stemmed from a single importation in August–December 2009. However, substantial increases in the incidence of acute flaccid paralysis (AFP) did not occur until early 2010. A vaccination campaign was launched in May and the outbreak abated after that. Figure 5A shows a time series of cases and sequences generated; the timing of the rounds of vaccination is also shown.

#### 3.3.1. Model

We modelled the transmission of poliovirus with a birth–death process with time-varying effective reproduction numbers and surveillance rates to explain the effect of vaccination and heightened surveillance once the outbreak was recognized.

We extracted the weekly case counts of laboratory-confirmed polio infections with paralysis [20] (with WebPlotDigitizer [26]) to use as $D_{\text{cases}}$, and obtained an alignment of publicly available sequences from Li *et al*. [15] (originally sequenced by Yakovenko *et al*. [19]) to use as $D_{\text{MSA}}$. We subtracted the number of sequences from the time series to avoid duplication. As part of the sensitivity analysis we re-ran the analysis without this subtraction step and obtained similar estimates (see electronic supplementary material, table S4.) We assume that, prior to the first sequence on 1 February 2010 the rate of sequencing and case observation probability was zero, and constant after that point. Further details of our model of surveillance, including the accession numbers for the sequences, are available in §7 of the electronic supplementary information.

Since case counts were only available at a weekly resolution, we distributed them uniformly across the days of the week and the sequenced samples uniformly within the date associated with them (when more than one genome was associated with the same date), i.e. cases were modelled as a daily time series of unsequenced samples and a point process of sequenced samples. Further details are available in §7 of the electronic supplementary information and electronic supplementary material, table S2 lists the prior distributions used in the model. The XML files specifying the full analysis and post-processing are available from https://github.com/aezarebski/timtam-tajikistan.

#### 3.3.2. Results

Figure 5B shows the estimates of the prevalence of infection and the 95% HPD intervals at 13 dates separated by 21-day intervals. Note that the estimates of the absolute prevalence extend before the first observed case. For example, we estimate that before February 2010, the prevalence was below 100. Even adjusting for a change in surveillance, there is little evidence of widespread transmission before February in the estimates of the prevalence of infection.

Figure 5C shows the estimates of the effective reproduction number through time along with the 95% HPD intervals. A full summary of parameter estimates can be found in electronic supplementary material, table S3. The estimates in figure 5C suggest the effective reproduction number may have already started to decline before the beginning of the vaccination rounds, potentially due to public awareness. A comparison of these estimates with previous age-structured estimates is given in electronic supplementary material, figure S4.

#### 3.3.3. Subsampling experiment

To investigate what information is provided by the time series data, we repeated the analysis described above using two different subsamples of the time series data. These subsamples of the poliomyelitis cases came from random samples of 33% and 66% of the daily case counts.

**Figure 6.**
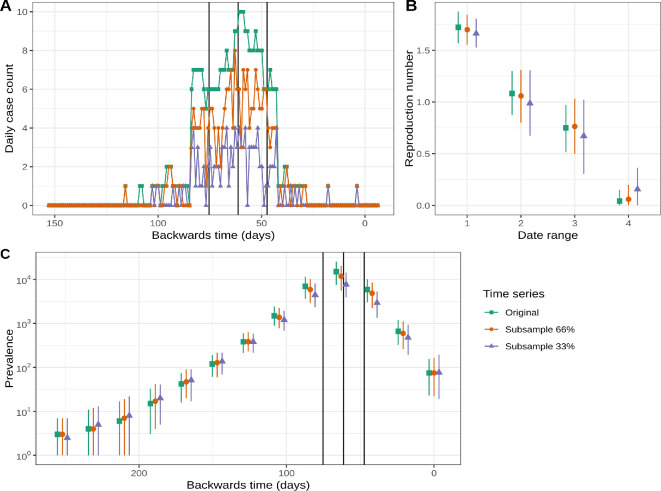
Using a subset of the time series data produces similar, though more uncertain, estimates of key epidemiological parameters. (A) The case counts (distributed across the days of the week) were randomly subsampled to keep approximately 66% and 33% of the cases. (B) The components of the piece-wise constant estimate of the reproduction number using the subsampled time series are similar to those obtained with the original time series but with a slight trend towards smaller values. The subsampled data estimates have wider HPD intervals. (C) The estimates of the prevalence through time are similar, however the estimates are smaller using the subsampled data. The black vertical lines show the change times of the reproduction number. Plots showing the estimates of the surveillance parameters are shown in electronic supplementary material, figure S9.

Figure 6A shows the two random subsamples of the time series of confirmed poliomyelitis cases used in the subsampling experiment. Figure 6B shows the estimates of the reproduction number through time given the subsampled time series. While the point estimates are similar (though slightly smaller) when using the subsampled time series, the HPD intervals are larger with the subsampled (smaller) time series. Figure 6C shows the prevalence through time using each of the subsampled time series. At the peak of the epidemic, the estimated prevalence is smaller when using the subsampled time series. However, during the early phase of the epidemic (when the only information stems from sequenced cases) prevalence estimates are slightly higher when using the subsampled time series.

Electronic supplementary material, figure S9 provides the estimates of the parameters of the surveillance system: the proportion of cases in the time series and the proportion sequenced. Estimates of the proportion of cases represented by the time series are inversely correlated to the amount of subsampling (as expected given we have subsampled this data). However, estimates of the proportion of cases sequenced are higher when using the subsampled time series.

## 4. Discussion

We implemented a model, Timtam, which can also act as a phylodynamic tree prior, to facilitate the co-estimation of the prevalence and the effective reproduction number. The model can draw on both sampled pathogen sequence data and an epidemic time series of confirmed cases (i.e. observations of infection for which the pathogen genome was not sequenced). The algorithm used to compute the (approximate) log-likelihood is fast, requiring a number of steps linear in the number of sequences and length of the time series of unsequenced cases [6]. Since the calculation time is proportional to the number of scheduled sampling times and not the number of cases in the epidemic time series, it is possible to use Timtam on epidemics with a large number of unsequenced cases. An implementation is available as a BEAST2 package and tutorials on the usage of the package are included with the source code: https://github.com/aezarebski/timtam2.

We extended our previous method [6] to estimate historical prevalence, by explicitly modelling prevalence as a model parameter. This differs from several previous approaches, in which estimates of the prevalence come from intermediate steps in the likelihood calculation or from *post hoc* simulation. Treating the prevalence as a bona fide parameter also means we can incorporate additional data concerning prevalence into the analysis. For example, if survey data on infection in a random sample from the population was available for specific dates (e.g. from seroprevalence surveys) we could condition the model on this as additional data.

We performed a simulation study to demonstrate that the method is well‐calibrated, i.e. that approximately 95% of the 95% HPD intervals contain the true value. The simulation study further demonstrated that the method’s performance does not degrade substantially when we aggregated the occurrence data into a time series, (the format it is usually found in). Considering how the error and uncertainty in the estimates depend upon the dataset size (in electronic supplementary material, figure S3), we recommend additional caution when applying Timtam to a dataset consisting of fewer than 150 confirmed cases.

We used the validated method to replicate two analyses of limited single-source outbreaks. The first, carried out by Andréoletti *et al*. [9], is of an outbreak of SARS-CoV−2 aboard the *Diamond Princess* cruise ship. The second empirical analysis, of the 2010 outbreak of poliomyelitis in Tajikistan, uses data from Yakovenko *et al*. [19] and the Centers for Disease Control and Prevention (CDC) [20].

The outbreak of SARS-CoV−2 aboard the *Diamond Princess* cruise ship was a relatively small, well-contained outbreak, for which the majority of infections were ascertained. However, only a small number of sequenced samples exist, all dating from a period of only three days (figure 4A). Our estimates of the reproduction number (displayed in figure 4C) are consistent with the values from Andréoletti *et al*. [9] and are broadly similar to those from Vaughan *et al*. [18]. However [18], only used genomic data, which may explain the difference in the $R_{\text{e}}$ estimates.

Our prevalence estimates are greater than those from Andréoletti *et al*. [9] (figure 4B). We attribute this difference to their implementation having an upper limit of 40 on the number of hidden lineages, which was necessitated by the computational complexity of the numerical integration algorithm used to compute the likelihood. As such, their estimates should be interpreted as lower bounds on the prevalence and not absolute estimates. Timtam overcomes this limitation by a negative binomial approximation of the number of hidden lineages, making it efficient at estimating large numbers of hidden lineages and applicable to real-world epidemic scenarios.

The empirical analysis of the 2010 outbreak of poliomyelitis in Tajikistan uses data from Yakovenko *et al*. [19] and the CDC [20]. This is a much larger outbreak over a period of months instead of weeks (figure 5A). We expect that most infections were not ascertained, since the majority of poliovirus infections are asymptomatic.

It is not possible to directly compare our estimates of the effective reproduction number of poliomyelitis in Tajikistan to previous work. Timtam does not support structured populations yet, so we were not able to obtain age-specific $R_{\text{e}}$ estimates such as those reported by Li *et al*. [15]. However, our estimates of the effective reproduction number during the central four weeks of the outbreak are similar to a demographically weighted average of the estimates from Li *et al*. [15]. In addition, Timtam allows us to obtain estimates of the outbreak prevalence through time. Our estimates suggest more than a hundred asymptomatic infections for every AFP case, which is consistent with previous estimates [27]. To the best of our knowledge, prevalence estimates for this outbreak have not been reported elsewhere.

Beginning with a phylodynamic analysis, it is interesting to consider what additional information, if any, is provided by the time series data (with this sampling model). To investigate this, we carried out an experiment in which we repeated the analysis with subsampled time series data. Removing approximately one or two-thirds of the time series data leads to slightly smaller estimates of the reproduction number and smaller estimates for the prevalence (although there is a substantial overlap in the credible intervals). Subsampling the time series increases the uncertainty of the estimates.

The estimates of the reproduction number and prevalence are reasonably robust to the subsampling, which we attribute to the genomic data being highly informative. We note that the estimated tree has many internal nodes extending back to the start of the epidemic. This insight into the dynamics early in the epidemic may explain why the genomic data is so informative in this case. In other situations, the time series data may be more informative.

When using the subsampled time series, as expected, the estimate of the proportion of infections observed decreases. However, the subsampling also led to larger estimates of the proportion sequenced. While unexpected, this is consistent with the smaller estimates of prevalence. Further work is needed to fully understand the potential for conflict in the information offered by sequence and time series data.

A limitation of our work is that it estimates the prevalence of infection, and not the incidence of infection, nor the cumulative incidence over the whole epidemic. That said, it is possible to obtain estimates of the net birth rate (i.e. rate of new infections) from the product of the prevalence and the birth rate. However, this is different from estimating the actual number of new infections that occurred in an interval, which is not possible with the current methodology.

In Bayesian phylodynamics, coalescent tree priors condition on the sampling times of sequence data. Birth–death tree priors can use information in sampling times but consequently are prone to bias if this aspect of the model is misspecified. Incorporating additional epidemiological data has been found to help when there are geographical biases in sequence sampling [28]. Understanding the intricacies of biased sampling is an active area of research; see, for example, the recent simulation studies investigating spatial [29] and temporal [30] bias.

Our implementation does not yet support the use of *sampled ancestors* [11], i.e. including a probability r that an infected individual is removed upon (unscheduled) observation. Extending the approximation to handle this case is feasible, however there are substantial software engineering challenges involved in implementing this in the BEAST2 platform. As the model and its implementation are useful without this extension we present it as is, and include the mathematical expressions required for including sampled ancestors in §4 of the electronic supplementary information.

In summary, the Timtam package is an efficient implementation of our model within the BEAST2 framework, where it can be combined with a multitude of other model components. The model is suitable as a tree prior, or demographic model, for unstructured outbreaks and provides similar functionality to the model presented by Andréoletti *et al*. [9], with the added advantage of being able to incorporate unsequenced cases (observations) as a time series, being able to condition on historical prevalence estimates and being efficient enough to handle large trees.

## Data Availability

The code used for the simulation study is available at [31]. The code and data used for the SARS-CoV-2 case study are available at [32]. The code and data used for the poliovirus case study are available at [33]. Supplementary material is available online [34].
